# Internalized weight bias and psychological wellbeing: An exploratory investigation of a preliminary model

**DOI:** 10.1371/journal.pone.0216324

**Published:** 2019-05-09

**Authors:** Morgan S. Lee, Brian D. Gonzalez, Brent J. Small, Joel Kevin Thompson

**Affiliations:** 1 University of South Florida, Tampa, FL, United States of America; 2 Moffitt Cancer Center, Tampa, FL, United States of America; Technion Israel Institute of Technology, ISRAEL

## Abstract

Although a growing body of literature demonstrates negative effects of internalized weight bias (IWB), the relationships between IWB and relevant social, psychological, and behavioral variables have not yet been evaluated systematically. The purpose of the present study was to create and assess a model of hypothesized risks and outcomes of IWB. In an online survey, 650 adult males and females completed self-report measures of IWB, self-esteem, weight-related stigma experiences, body-related shame, body satisfaction, societal influence on body image, appearance comparisons, binge eating, distress, and weight-related quality of life. The originally hypothesized model did not provide an adequate fit to the data. Iterative modifications were undertaken, and the resulting model, in which social factors were associated with IWB and body image-related constructs which were in turn associated with psychological and behavioral outcomes, provided excellent fit to the data (CFI > .99, SRMR = .02, and RMSEA = .03). Most model paths were similar for underweight or normal weight participants versus participants with overweight or obesity. This study represents an initial effort at constructing a comprehensive model of IWB that can be further refined in future research and used to help guide the development of related interventions.

## Introduction

Amidst alarm about high rates of overweight and obesity, awareness is growing of the prejudiced attitudes and discriminatory practices individuals with obesity often face, and empirical evidence indicates that this stigma has increased over time [1]. Weight bias/obesity stigma appears to be one of the few remaining acceptable forms of discrimination, and it occurs across numerous life domains including work, education, interpersonal relationships, health care, customer service, and the media [2,3]. Furthermore, the literature on weight bias interventions indicates these negative attitudes and beliefs are difficult to change, with most interventions producing only a small amount of reduction in bias [4]. Although formal models of weight bias are only in early stages of development (e.g., the COBWEBS model [5]), a sizable body of research has revealed that weight bias negatively impacts the psychological, behavioral, and physical health of its targets, leading to outcomes such as decreased mood and self-esteem, heightened body dissatisfaction, avoidance of exercise, and disordered eating behaviors [3]. However, less is known about the impact of self-stigma in the context of body weight.

While weight bias refers to negative attitudes and beliefs about other people on the basis of body weight, internalized weight bias (IWB) refers to self-directed stigmatizing attitudes people hold based on social stereotypes about their perceived weight status [6]. IWB is related to but distinct from body image: IWB emphasizes beliefs in social stereotypes about weight status, while body image refers to a multidimensional (i.e., based on other features in addition to body weight) evaluation of one’s body [7]. Much is now known about other-focused weight bias [2,3]; however, the potential risks and outcomes of IWB have only recently begun to garner substantial attention in research, aided by the creation and validation of measures to assess IWB (e.g., [6,8]) that have rendered the construct more accessible as a topic of study.

Early research on IWB has been conducted primarily in clinical settings with participants seeking treatment for weight loss (e.g., [9]) or eating disorders (e.g., [10]), although a few studies have used samples from general community settings (e.g., [11]). The existing body of research indicates that IWB is associated with negative psychological outcomes including reduced quality of life (e.g., [10,12]), symptoms of depression and anxiety [9,11], low self-esteem [6], and poor body image (e.g., [6,9]); negative physiological outcomes including elevated triglycerides and increased risk for metabolic syndrome [13]; and behavioral problems including disordered eating behaviors (e.g., [10,14]) and avoidance of exercise (e.g., [15]). However, the relationships between IWB and relevant social, psychological, and behavioral predictor and outcome variables have not yet been evaluated systematically.

**Fig 1:**
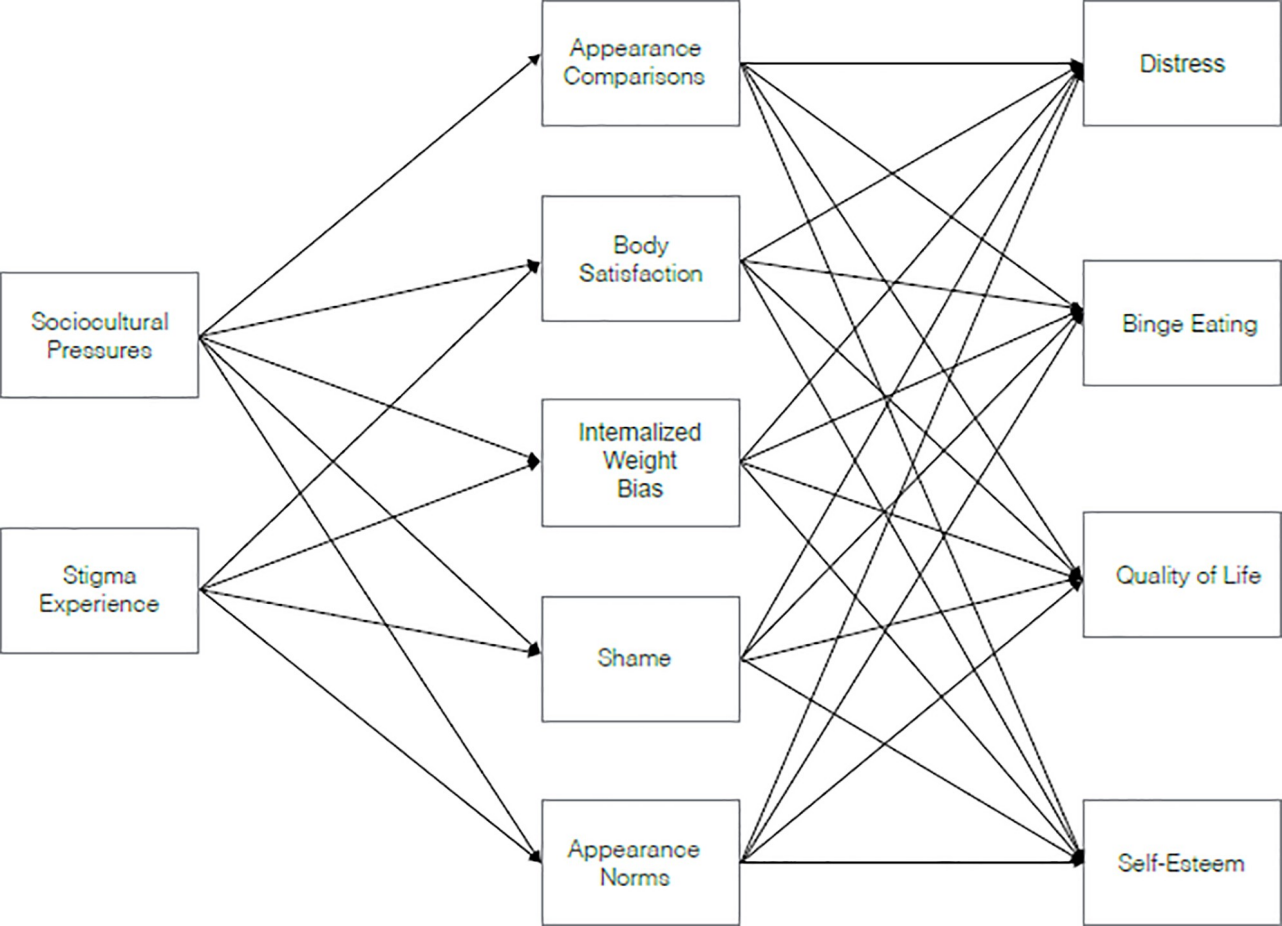
Original hypothesized model.

Given the significance of IWB in understanding and potentially alleviating distress across several domains, the risks and outcomes IWB should be explored. Aspects of one body image model, the Tripartite Model [16,17], may provide a starting point for some hypothetical connections. The Tripartite Model postulates that peers, parents, and the media influence body image and eating disturbances through the key mechanisms of appearance comparison and internalization of the thin ideal; the eating disturbances that result from these processes are subsequently hypothesized to impact psychological functioning. Specific to the current model (see Fig 1), the influences component was hypothesized to serve as a risk for IWB (i.e., negative feedback regarding one’s weight would have an impact on one’s level of IWB). Weight-based stigmatization was also deemed likely to be a risk for IWB given that being the target of weight-based stigmatization serves as an intense, personal form of exposure to negative stereotypes about overweight and obesity and that fear of enacted stigma (i.e., actual experience of discriminatory social actions) is recognized as a component of IWB [8].

At the next level of the proposed model (see Fig 1), several mediators were hypothesized to link level one risks to negative outcomes. Specifically, appearance comparisons, internalization of appearance norms, and body dissatisfaction (from the Tripartite Model) as well as body shame and internalization of weight-based stereotypes (based on prior literature [18,19]), were evaluated at this level. Placing the constructs of body dissatisfaction and body shame at level two or three was a difficult decision. In the Tripartite Model, body image is conceptualized as an outcome of level one influences and level two internalization and comparison. However, given that the proposed model also had multiple behavioral and psychological outcomes as components of level three (see Fig 1), which have mainly been tested with body image measures serving as risks for these outcomes (not as concurrent final level outcomes), we decided to leave body image measures in level two (with some exploratory testing of a four-level model as noted in the Statistical Analyses section below).

Proceeding to the final level of the proposed model (see Fig 1), the empirical literature on IWB (discussed above) has tied this construct to a number of negative outcomes. Some of the most consistent associations have been with distress [9,11]; eating behavior, particularly binge eating [9,14]; self-esteem [6]; and quality of life [10,12]. Consequently, these constructs were positioned as outcomes in the proposed model.

Most IWB research has been conducted in samples of participants whose BMI meets at least the overweight cutoff. However, some evidence suggests that perceived weight status is equally important as body mass index (BMI) in predicting psychosocial outcomes [20], and IWB has been found to be predictive of eating disorder pathology in lean (i.e., normal or underweight based on BMI) adults [21]. Accordingly, the present study included participants of all weight statuses so that possible differences in the relationships between IWB and other variables based on BMI-derived weight status could be explored. In sum, this cross-sectional study sought to examine potential risks and outcomes of IWB in an effort to serve as a basis for additional research and to reveal points at which intervention might be appropriate and effective.

## Materials and methods

### Participants and procedure

Study participants were male and female students aged 18 years and older who were recruited through the online participant pool of a large public university in the southeastern United States. No BMI restrictions were applied; participants of all weight statuses were recruited. Data were collected from 652 participants. Two participants provided height and weight data that would be unsustainable for adult human life, making it impossible to accurately classify their BMI-based weight status; these cases were deleted, so 650 cases were retained for the present analyses. Characteristics of the study participants (the sample overall and U/N and O/Ob participants separately) are displayed in Table 1. In the sample overall, participants were mostly female (81%), non-Hispanic (77%), Caucasian (72%), and heterosexual (91%), with a mean age of approximately 22 years old and a mean BMI of approximately 24. Participants came from all class years in college, with greater representation of juniors and seniors than freshmen and sophomores. Two-thirds of participants perceived their weight as “average,” and just over one-quarter (27%) perceived themselves as “overweight” or “very overweight.” Participants completed validated self-report questionnaires in an online survey that took approximately 45 minutes to finish, and they were subsequently compensated with course credit. The study protocol was approved by the University of South Florida Institutional Review Board, and all participants provided written consent in a digital format.

**Table 1:** Demographic, psychological, and behavioral characteristics of study participants.

	Total Sample (*N* = 650)	U/N (*n* = 443)	O/Ob (*n* = 207)
Gender (% female)	528 (81%)	364 (82%)	164 (79%)
Age *M* (range)	21.75 (18–54)	21.29 (18–46)	22.75 (18–54)
BMI *M* (range)	24.07 (16–61)	21.49 (16–25)	29.58 (25–61)
Ethnicity (% non-Hispanic)	499 (77%)	340 (77%)	159 (77%)
Race (% Caucasian)	463 (72%)	319 (73%)	144 (70%)
Sexuality (% heterosexual)	593 (91%)	410 (93%)	183 (88%)
Education^a^			
Freshman	121 (19%)	94 (21%)	27 (13%)
Sophomore	110 (17%)	77 (17%)	33 (16%)
Junior	193 (30%)	134 (30%)	59 (29%)
Senior	192 (30%)	115 (26%)	77 (37%)
Perceived Weight			
Very Underweight	3 (<1%)	3 (1%)	0 (0%)
Underweight	40 (6%)	38 (9%)	2 (1%)
Average	427 (66%)	363 (82%)	64 (31%)
Overweight	151 (23%)	37 (8%)	114 (55%)
Very Overweight	29 (4%)	2 (<1%)	27 (13%)

Note. U/N = underweight/normal weight. O/Ob = overweight/obese.

^a^Percentages do not add to 100 because a small number of participants responded “other” for their year in college.

### Measures

#### Demographics

A standard self-report questionnaire was used to obtain participant characteristics including gender, age, year in college, race, ethnicity (*Hispanic* or *non-Hispanic*), sexual orientation, height, weight, and perceived weight status. For the perceived weight status item, participants were asked to describe their weight as one of the following: *Very Underweight*, *Underweight*, *Average*, *Overweight*, and *Very Overweight*. For the present analyses, participants’ BMI (obtained from their self-reported height and weight) was used to form underweight/normal weight (U/N; BMI ≤ 25) and overweight/obese (O/Ob; BMI ≥ 26) groups.

#### IWB

The Weight Bias Internalization Scale (WBIS) [6] was used to assess IWB. The WBIS contains 11 items rated on a 7-point Likert scale ranging from 1 (*Strongly Disagree*) to 7 (*Strongly Agree*). Two items are reverse-scored, and the mean of all the item responses is used as the participant’s score, with higher scores indicating greater IWB. The WBIS demonstrated satisfactory psychometric properties in its initial validation study [6]. Cronbach’s alpha for the measure in the present study was .86 for U/N participants and .91 for O/Ob participants.

#### Self-esteem

Self-esteem was assessed using the Rosenberg Self-Esteem Scale (RSES) [22], which contains 10 items rated on a 4-point Likert scale ranging from 0 (*Strongly Disagree*) to 3 (*Strongly Agree*). Half of the items are reverse-coded, and the sum of all the item responses is used as the participant’s score, with higher scores indicating higher self-esteem. Cronbach’s alpha for the measure in the present study was .91 for U/N participants and .88 for O/Ob participants.

#### Stigma experiences

Weight-based stigma experiences were evaluated using the Stigmatizing Situations Inventory (SSI) [23]. The SSI contains 50 items that assess the frequency of various stigmatizing experiences on a scale of 0 (*Never*) to 9 (*Daily*). For the current analyses, the mean of all the item responses was used as the participant’s score, with higher scores indicating more experiences with weight-based stigma. Cronbach’s alpha for the measure in the present study was .98 for both U/N and O/Ob participants.

#### Shame

Body-related shame (i.e., feelings of shame induced by perceived nonconformity to standards for weight-related behavior and appearance) was assessed using the Body Shame Scale of the Objectified Body Consciousness Scale (OBCS-Shame) [24]. The OBCS-Shame contains eight items rated on a 7-point Likert scale ranging from *Strongly Disagree* to *Strongly Agree*. The mean of all the item responses is used as the participant’s score, with higher scores indicating more body-related shame. Cronbach’s alpha for the measure in the present study was .85 for both U/N and O/Ob participants.

#### Body satisfaction

Body satisfaction was evaluated using the Appearance Evaluation Subscale (AES) and Body Areas Satisfaction Scale (BASS) of the Multidimensional Body-Self Relations Questionnaire (MBSRQ) [25]. The AES contains seven self-evaluative statements about one’s own body that participants rate on a 5-point Likert scale ranging from 1 (*Definitely Disagree*) to 5 (*Definitely Agree*). The BASS contains nine items that ask participants to indicate their satisfaction/dissatisfaction with various aspects of their bodies using a 5-point Likert scale ranging from 1 (*Very Dissatisfied*) to 5 (*Very Satisfied*). For the current analyses, the mean of the AES and BASS was used as participants’ body satisfaction score, with higher scores indicating greater body satisfaction. Cronbach’s alpha for the measure in the present study was .93 for both U/N and O/Ob participants.

#### Appearance norms and sociocultural pressures

Societal influence on body image was assessed using the Sociocultural Attitudes Towards Appearance Scale– 4 (SATAQ– 4) [26]. The SATAQ– 4 contains five factors assessing two main concepts: internalization of societal body ideals (i.e., appearance norms; two factors: thin/low body fat and muscular/athletic) and appearance-related pressures (i.e., sociocultural pressures; three factors: family, peers, and media). Participants are asked to rate each item on a scale of 1 (*Definitely Disagree*) to 5 (*Definitely Agree*); higher scores indicate more internalization and appearance-related pressures. A pre-publication 28-item version of this measure was administered to participants; however, the final published version of the measure contains just 22 items. For study analyses, additional items that had been administered were excluded; however, two items from the muscular/athletic subscale of general internalization were phrased slightly differently from the phrasing used in the final measure. Nonetheless, Cronbach’s alpha for this subscale was identical to that found in the final measure’s validation article (for the overall sample in each study, α = .91). In the present study, Cronbach’s alpha for appearance-related pressures was .92 for U/N participants and .91 for O/Ob participants; Cronbach’s alpha for internalization of societal body ideals was .87 for U/N participants and .88 for O/Ob participants.

#### Appearance comparisons

Participants’ tendency to compare their own physical appearance to the physical appearance of other people was evaluated using the Physical Appearance Comparison Scale (PACS) [27]. The PACS contains five items about the frequency of appearance-related comparisons in social situations; participants are asked to rate each item on a 5-point Likert scale ranging from 1 (*Never*) to 5 (*Always*). The mean of all the item responses is used as the participant’s score, with higher scores indicating more appearance comparison. Cronbach’s alpha for the measure in the present study was .79 for U/N participants and .75 for O/Ob participants.

#### Binge eating

Binge eating was assessed using the Binge Eating Scale (BES) [28], a 16-item measure that addresses behavioral, cognitive, and affective aspects of binge eating. In each item, participants are given a set of four statements (each of which has a designated point value for scoring purposes) and are asked to choose which statement best describes their control over their own eating behavior. The total number of points is used as the participant’s score, and cutoffs are used to classify participants’ behavior as non-binging, moderate binging, and severe binging (higher scores indicate more binge eating symptomatology). Cronbach’s alpha for the measure in the present study was .89 for U/N participants and .91 for O/Ob participants.

#### Distress

Distress was evaluated using the 21-item short form of the Depression Anxiety Stress Scales (DASS) [29], which assesses three aspects of negative emotion: depression, anxiety, and tension/stress. For each item, participants are asked to rate the mood-related statement on a scale of 0 (*did not apply to me at all*) to 3 (*applied to me very much*, *or most of the time*) with respect to their mood in the past week. For the current analyses, the mean of all item responses was used as the participant’s score, with higher scores indicating more negative emotional symptoms. Cronbach’s alpha for the measure in the present study was .95 for U/N participants and .96 for O/Ob participants.

#### Quality of life

Weight-related quality of life was evaluated using the Impact of Weight on Quality of Life–Lite (IWQOL-Lite) [30]. The IWQOL-Lite contains 31 items comprising five scales: physical function, self-esteem, sexual life, public distress, and work. Participants are asked to rate each item on a 5-point Likert scale ranging from 1 (*Never True*) to 5 (*Always True*) with regard to the impact of their weight on their daily life in the past week. For the current analyses, the standardized total score was used as the participant’s score, with higher scores indicating better weight-related quality of life. Cronbach’s alpha for the measure in the present study was .95 for both U/N and O/Ob participants.

### Statistical analyses

Before conducting the main analyses, descriptive statistics were produced for participant characteristics and model variables. Next, correlations between proposed model variables were calculated. Models were tested using Mplus version 7.4 [31]. First, a base model with three levels was tested on the basis of *a priori* hypotheses of associations between risks, IWB and associated attitudes, and outcomes (see Fig 1). A model with four levels that was more similar to the structure of the Tripartite Model was also explored, but this model provided substantially poorer fit to the data compared to the originally hypothesized three-level model; thus, the original model was retained for the remainder of the analyses. Next, this model was refined by first deleting those paths that were not statistically significant (with statistical significance set at *p* ≤ .05). Paths were added to the model based on modification indices, which suggest additional paths that can significantly enhance model fit. Suggested paths were required to conform to current theoretical understanding of the constructs involved and to improve the -2 log likelihood of the model by at least 10 points to be considered for inclusion. Finally, paths of participants who were underweight or within normal weight range (BMI ≤ 25) were computed separately from those of participants with overweight or obesity (BMI ≥ 26). Model fit was determined using the comparative fit index (CFI), root mean square error of approximation (RMSEA), and standardized root mean square residual (SRMR). CFI values ≥ .95 and SRMR values < .08 indicate good model fit [32]. RMSEA values ≤ .05, between .05 and .08, and between .081 and .10 indicate good, acceptable, and marginal model fit, respectively [33].

## Results

**Fig 2:**
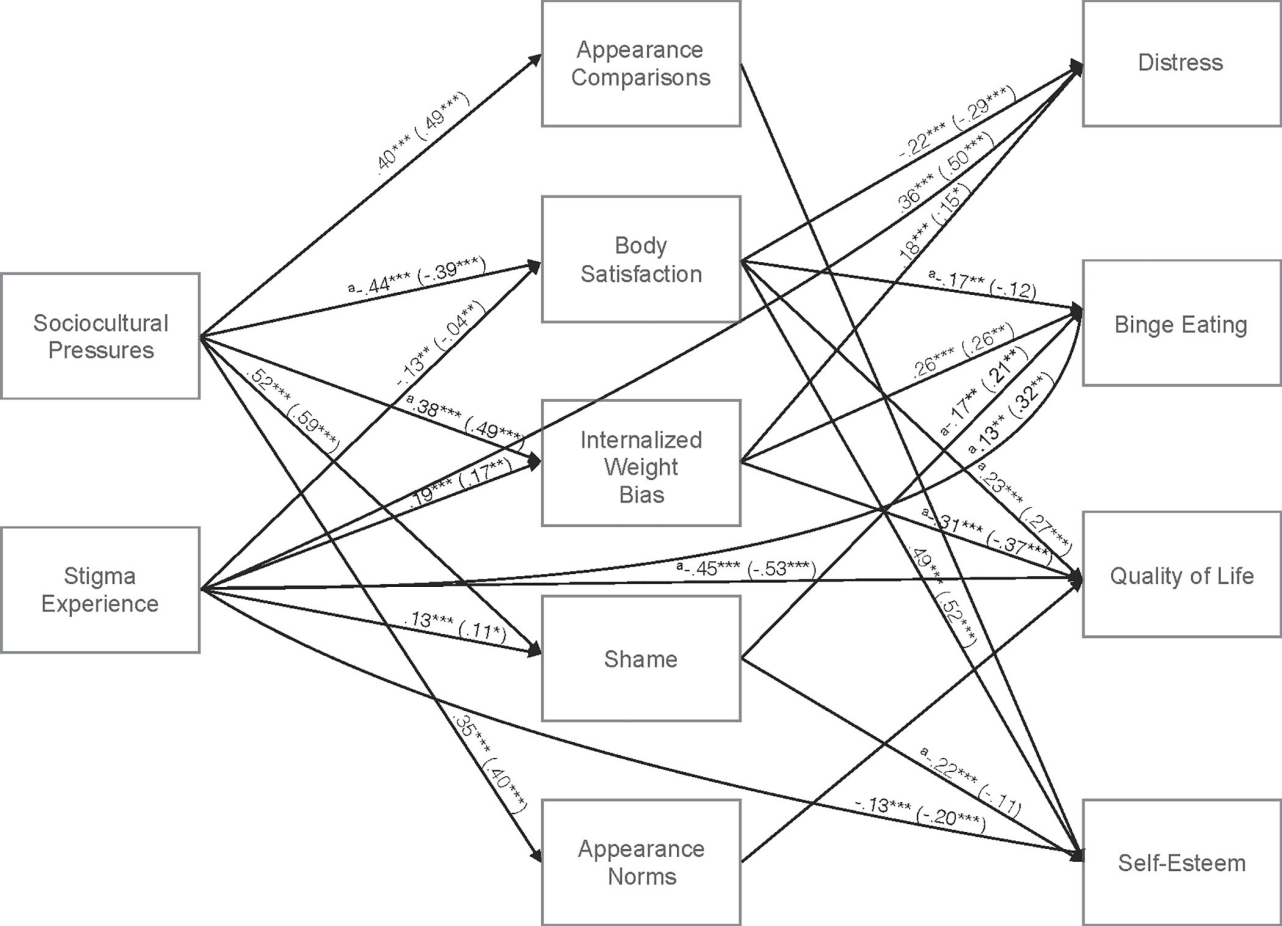
Final model. U/N values outside parentheses; O/Ob values inside parentheses. ^a^U/N path value differs significantly from O/Ob path value. ***p < .001. ** *p* < .01. *p < .05.

Table 2 provides descriptives and correlations for model constructs, both for the sample as a whole and by group. All correlations were significant, with the exception of the correlation between appearance norms and stigma experience. The original theorized model (see Fig 1) did not provide a good fit to the data, with CFI = .91, SRMR = .07, and RMSEA = .26. After five iterative modifications to the model (see Table 3), the resulting model (see Fig 2) provided excellent fit to the data, with CFI > .99, SRMR = .02, and RMSEA = .03. Covariances between variables at each level were retained in the model but are not included in the figure to enhance legibility; nonsignificant paths were omitted from the figure for the same reason. Paths from sociocultural pressures to appearance comparisons and from stigma experiences to appearance norms were removed from the final model. Paths from appearance comparisons to binge eating, quality of life, and self-esteem were also removed. Paths from stigma experience to self-esteem and from shame to distress and quality of life were removed as well. Lastly, paths from appearance norms to distress, binge eating, and self-esteem were removed from the model. Paths were added to the model from stigma experience to distress, binge eating, quality of life, and self-esteem. Thus, stigma experience had indirect effects on these outcomes through the mediators but also direct effects. In the final model, sociocultural pressures demonstrated stronger relationships with level two variables than did stigma experience, although (as noted above) stigma experience was also directly related with level three outcomes variables, whereas sociocultural pressures was not. Additionally, the level two variables body satisfaction, IWB, and (to a somewhat lesser degree) shame demonstrated more and stronger relationships with outcomes than did appearance comparisons and norms.

**Table 2:** Descriptives and correlations for model constructs.

Construct	Total Sample *M*(SD)	U/N *M*(SD)	O/Ob *M*(SD)	Correlations (below diagonal = total sample; above diagonal = U/N and O/Ob groups respectively)										
				1	2	3	4	5	6	7	8	9	10	11
1. Sociocultural Pressures	2.61 (0.97)	2.40 (0.93)	3.07 (0.91)		.20/.28	.40/.49	-.47/-.40	.42/.54	.59/.61	.35/.40	.27/.35	.35/.39	-.34/-.43	-.30/-.34
2. Stigma Experience	0.60 (1.04)	0.45 (0.91)	0.93 (1.22)	.28		.11/.26	-.22/-.17	.27/.32	.24/.29	.05^b^/.07^b^	.46/.61	.28/.61	-.58/-.68	-.44/-.35
3. Appearance Comparison	2.91 (0.79)	2.88 (0.80)	2.96 (0.77)	.42	.17		-.36/-.40	.37/.55	.47/.55	.45/.49	.27/.41	.29/.41	-.26/-.43	-.36/-.40
4. Body Satisfaction	3.41 (0.79)	3.58 (0.73)	3.06 (0.81)	-.50	-.25	-.37		-.57/-.73	-.58/-.64	-.24/-.35	-.42/-.50	-.45/-.50	.49/.60	.67/.65
5. Stigma Internalization	3.10 (1.33)	2.82 (1.17)	3.68 (1.47)	.51	.33	.42	-.67		.60/.77	.34/.51	.43/.58	.49/.61	-.53/-.70	-.51/-.64
6. Shame	3.44 (1.32)	3.23 (1.29)	3.86 (1.29)	.62	.29	.49	-.62	.68		.46/.50	.40/.51	.49/.56	-.47/-.55	-.58/-.55
7. Appearance Norms	3.19 (0.80)	3.21 (0.78)	3.16 (0.83)	.34	.05^a^	.46	-.26	.38	.45		.15/.27	.28/.29	-.13^c^/-.26	-.21/-.25
8. Mood	0.53 (0.58)	0.49 (0.54)	0.61 (0.63)	.31	.53	.32	-.46	.50	.45	.19		.51/.55	-.57/-.71	-.49/-.63
9. Binge Eating	8.97 (8.17)	7.41 (7.16)	12.30 (9.17)	.42	.41	.33	-.51	.58	.54	.26	.53		-.55/-.72	-.37/-.49
10. Quality of Life	88.04 (14.52)	91.77 (11.63)	80.06 (16.73)	-.44	-.65	-.31	.59	-.65	-.53	-.16	-.62	-.67		.46/.58
11. Self-Esteem	21.10 (5.57)	21.38 (5.59)	20.50 (5.51)	-.41	-.32	-.38	.65	-.55	-.57	-.22	-.54	-.41	.49	

Note. U/N = underweight/normal weight. O/Ob = overweight/obese. Unless otherwise noted, all *p*-values for correlations are significant at *p* < .001.

^a^*p* = .19.

^b^*p* = .28.

^c^*p* = .005.

**Table 3:** Fit statistics for path analysis models.

Model Number	CFI	RMSEA	SRMR
1	.91	.26	.07
2	.91	.20	.07
3	.91	.19	.07
4	.98	.09	.04
5	>.99	.03	.02

Note. CFI = Comparative fit index. RMSEA = Root mean square error of approximation. SRMR = standardized root mean square residual. CFI values ≥ .95 and SRMR values < .08 indicate good model fit. RMSEA values ≤ .05, between .05 and .08, and between .081 and .10 indicate good, acceptable, and marginal fit, respectively.

The final analysis compared path estimates between participants whose BMI indicated underweight or normal weight (BMI < 25) and those whose BMI indicated overweight or obesity (BMI ≥ 25). In this analysis, paths from appearance comparisons to self-esteem, from appearance comparisons to distress, and from appearance norms to quality of life were no longer significant for either group (*p*s > .05). A significant path remained from body satisfaction to binge eating for underweight or normal weight participants, such that greater body satisfaction was associated with less binge eating behavior (*p* < .01). However, among participants with overweight or obesity this association was no longer significant (*p* > .05).

## Discussion

The present study aimed to provide structure to the literature on IWB, creating a model of risks and outcomes of IWB that can both facilitate future work exploring mediators and moderators of IWB’s relationships with other constructs and inform intervention design. Although nearly all model constructs were significantly correlated, the original model did not provide an adequate fit to the data. After five iterative modifications to the model, a model with excellent fit and a similar structure as the originally proposed model was produced.

Despite not being included in the original model, the paths that were later added from stigma experience to the outcomes of distress, binge eating, quality of life, and self-esteem, paths which indicate that stigma experience has direct effects on these outcomes in addition to the hypothesized indirect effects, are not surprising. Weight-based stigma has been associated with such outcomes in other studies (for a review, see [3]), and the present results indicate it works in both direct and indirect ways to produce harm. The findings also suggest that the relationships between constructs in the model are largely independent of weight status, as the paths between constructs were similar between participants with BMIs in the underweight or normal categories and participants with BMIs in the overweight or obese categories (with a few exceptions where statistically significant differences were observed in the strength of a path in one group versus another; see Fig 2).

The relative strength of the paths retained in the model is noteworthy. Although both sociocultural pressures and stigma experience were significantly related to level two variables, the paths from sociocultural pressures were notably stronger. This suggests that sociocultural pressures may play a larger role than specific stigmatizing experiences in the internalization of weight bias and development of body-related shame and dissatisfaction, at least among university students, who comprised the sample in this study. It is unclear whether this pattern would emerge in clinical samples of individuals seeking treatment for obesity. Additionally, measurement of stigmatizing experiences has received relatively little attention in the existing psychometric literature and thus may not have been captured particularly well here, although the study employed the most prominent validated measure of the construct. Also notable is that in the final model, appearance comparisons and appearance norms were not strongly related to any of the outcomes. Body dissatisfaction, IWB, and to a lesser extent body-related shame, appear to be more influential in producing negative outcomes.

Strengths of the present study include its large sample size and its theory-driven, comprehensive approach to building the model of IWB. The study also had limitations including its cross-sectional design and use of self-report measures. Additionally, the sample of college students used in this study represents a critical population in the context of IWB and body dissatisfaction but is relatively homogenous and may not be representative of all adults who are impacted by IWB, particularly with respect to disordered eating behaviors such as the outcome of binge eating in the present study. Also, this study used path analysis because each of the constructs was assessed with only a single scale. Future studies could include multiple indicators and use structural equation modeling, a more robust analytic tool. Finally, the measure of IWB used in this study (the WBIS) has traditionally been used in exclusively overweight/obese samples. A newer version of the WBIS designed for use with participants of all weight statuses is now available [34] but was not published at the time this study was designed and conducted. However, the original WBIS has been used successfully in samples that include participants with BMIs that do not meet cutoffs for overweight or obesity (e.g., [21,35]), a recent analysis of the psychometric properties of the WBIS indicates the measure functions similarly across the weight spectrum [36], and paths from the WBIS to other model variables were largely similar in both weight groups in the present findings.

Future research should explore the relationships in this model using prospective designs and in other populations to ensure the robustness of the model. Additionally, following in the footsteps of body image-related work using the Tripartite Model, the relationships in this model could be used to drive work on development of interventions for IWB. The varying strengths of the paths in the final model are instructive, suggesting which constructs may be particularly useful intervention targets. For example, the present results suggest that sociocultural pressures might be a more rewarding target than would stigmatizing experiences when the goal is to alleviate IWB and related concerns such as body dissatisfaction. Such efforts could focus on the pressures themselves (e.g., advocating for more diverse portrayals of beauty and fitness in the media) or on individuals’ perceptions of those pressures (e.g., media literacy training). The present study represents the first efforts at building a comprehensive model of IWB that can be further refined in future research and used to help guide the development of related interventions.
